# Suspected COVID-19–Induced Myopericarditis

**DOI:** 10.31486/toj.20.0090

**Published:** 2021

**Authors:** Ivana Okor, Amber Sleem, Alice Zhang, Rikin Kadakia, Tamunoinemi Bob-Manuel, Selim R. Krim

**Affiliations:** ^1^ Department of Hospital Medicine, Ochsner Clinic Foundation, New Orleans, LA; ^2^ The University of Queensland Faculty of Medicine, Ochsner Clinical School, New Orleans, LA; ^3^ Department of Cardiology, John Ochsner Heart and Vascular Institute, Ochsner Clinic Foundation, New Orleans, LA

**Keywords:** *Cardiomyopathies*, *COVID-19*, *myocarditis*, *pericarditis*, *Takotsubo cardiomyopathy*

## Abstract

**Background:** The incidence of myocarditis in patients with coronavirus disease 2019 (COVID-19) remains unknown; however, increasing evidence links COVID-19 to cardiovascular complications such as arrhythmias, heart failure, cardiogenic shock, fulminant myocarditis, and cardiac death. We present a case of suspected COVID-19–induced myopericarditis and discuss the diagnostic implications, pathophysiology, and management.

**Case Report:** A 72-year-old female was admitted to the hospital with acute on chronic respiratory failure in the setting of COVID-19. The next day, she developed pressure-like retrosternal chest pain. Laboratory findings revealed elevated cardiac enzymes and inflammatory markers consistent with myocardial injury. Electrocardiogram revealed diffuse ST segment elevations without reciprocal changes, concerning for myopericarditis. Transthoracic echocardiography showed new findings of severely reduced left ventricular (LV) systolic function, with an estimated ejection fraction (EF) of 20%. Her hospital course was further complicated by cardiogenic shock that required treatment in the intensive care unit with vasopressors and inotropes. During the next few days, she had almost full recovery of her LV function, with EF improving to 50%. However, her clinical status deteriorated, likely the result of a bowel obstruction. She was transitioned to comfort care at the request of her family, and she died shortly after.

**Conclusion:** This case highlights diagnostic and therapeutic challenges that physicians may encounter when managing acute cardiac injury in the setting of COVID-19. The multiple mechanisms of COVID-19–related myocardial injury may influence the approach to diagnosis and treatment.

## INTRODUCTION

As of September 2020, coronavirus disease 2019 (COVID-19) had affected more than 30 million people globally.^1^ COVID-19–related cardiovascular complications such as tachyarrhythmia, heart failure, cardiogenic shock, fulminant myocarditis, and cardiac death have been reported.^2^ The pathophysiologic mechanisms responsible for COVID-19–induced myocarditis remain under investigation. We present a case of suspected COVID-19–induced myopericarditis and discuss the diagnostic implications, pathophysiology, and management.

## CASE REPORT

A 72-year-old female with a medical history of systemic hypertension, chronic obstructive pulmonary disease (COPD) on 2 L of oxygen (O_2_) via nasal cannula presented to the emergency department (ED) with worsening shortness of breath after being diagnosed 1 week earlier with COVID-19. On arrival in the ED, she was afebrile but tachycardic, with a heart rate of 132/min. Blood pressure was 145/80 mmHg, and O_2_ saturation was 98% on 15 L non-rebreather mask. Initial laboratory tests revealed a normal leukocyte count but elevated acute phase reactant markers, including C-reactive protein (CRP) of 35.1 mg/L (reference range, 0-8.2 mg/L), D-dimer of 0.51 mg/L FEU (reference range, <0.5 mg/L FEU), and lactate dehydrogenase (LDH) of 379 U/L (reference range, 110-260 U/L).

The patient was admitted for hypoxic respiratory failure. Initial therapy included 500 mg oral azithromycin and 1 g intravenous (IV) ceftriaxone daily. She also received 1 dose of 125 mg IV methylprednisolone, followed by 40 mg of oral prednisone daily.

**Figure 1.**
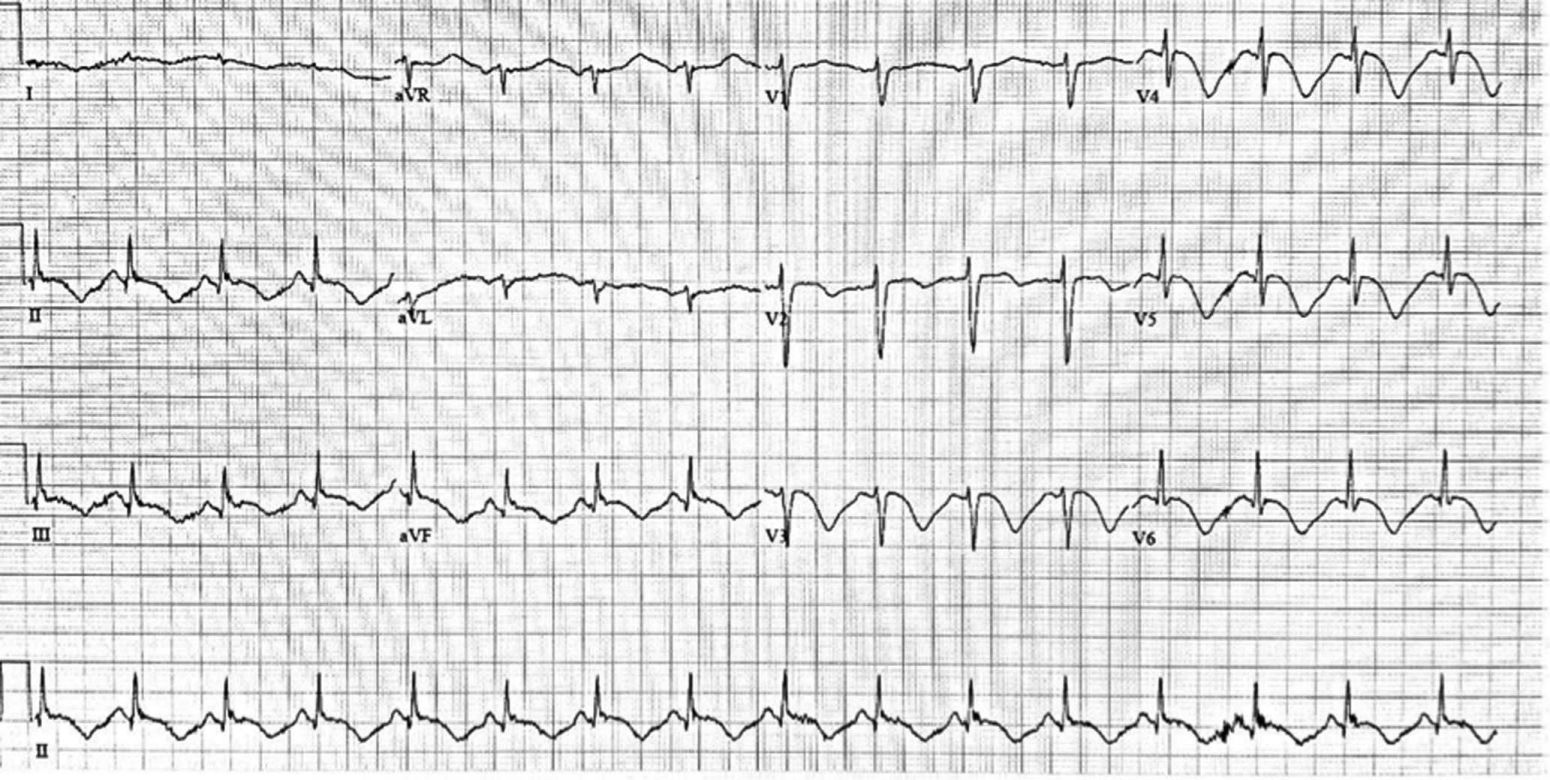
Initial electrocardiogram with normal sinus rhythm shows T wave inversions in inferior and anterolateral leads with prolonged QTc.

On hospital day 2, she developed a pressure-like retrosternal chest discomfort that radiated to her shoulders. Electrocardiogram (ECG) showed diffuse T wave inversions in the inferior and anterolateral leads with prolonged QTc 660 ms (Figure 1). Serial cardiac enzymes showed up-trending of high-intensity troponin markers from 0.01 to 1.0 ng/mL. Because of acute ECG changes and elevated cardiac enzymes, the patient was empirically treated with oral aspirin 325 mg, ticagrelor 180 mg, and a heparin infusion (initial bolus 60 U/kg, followed by 12 U/kg/h).

**Figure 2.**
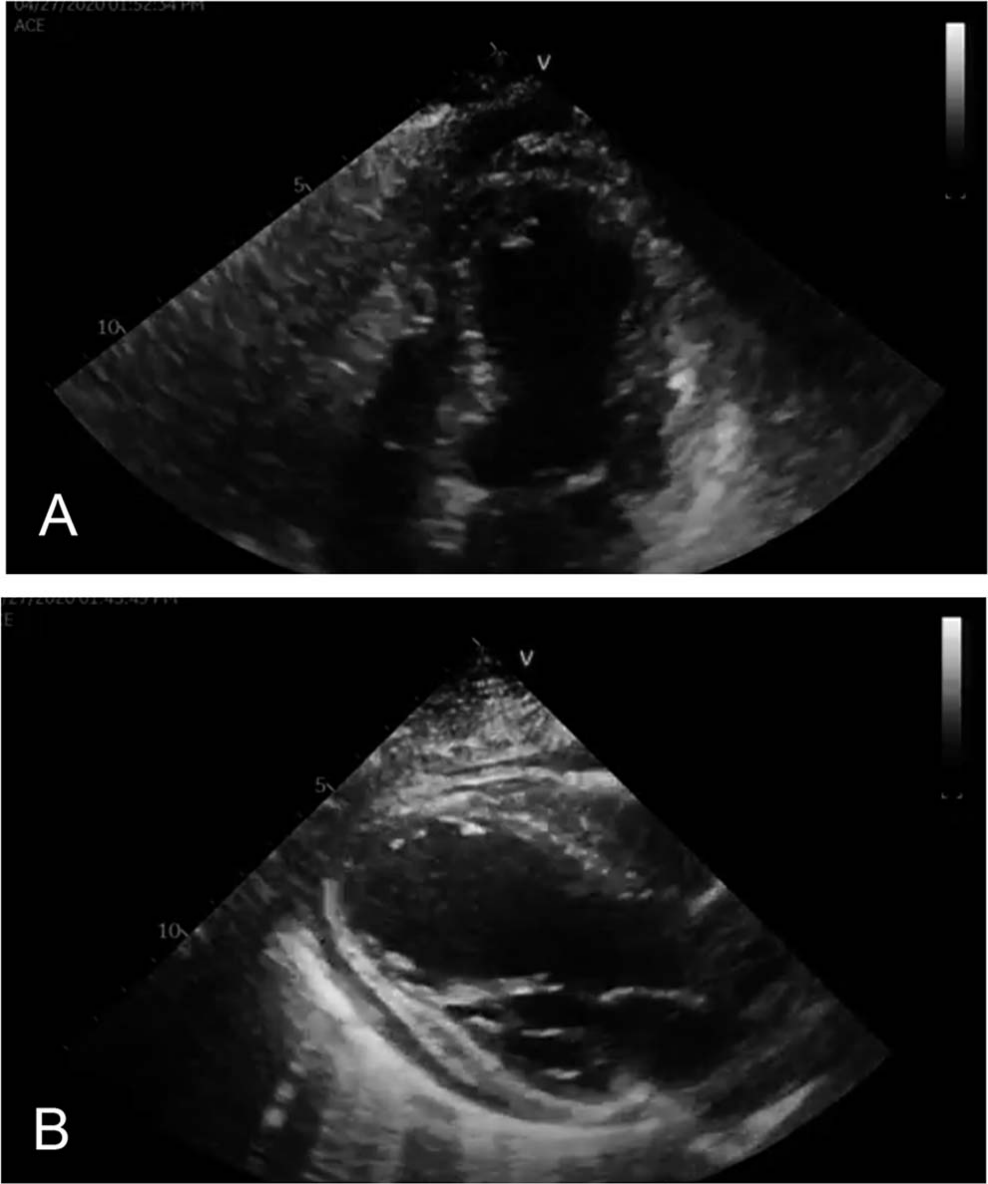
Initial echocardiogram in (A) 4-chamber view shows multiple segmental abnormalities and an ejection fraction of 20%. (B) Parasternal long axis view shows multiple segmental abnormalities, trace effusion, and low ejection fraction of 20%.

On hospital day 3, transthoracic echocardiography (TTE) showed new findings of severely reduced left ventricular (LV) systolic function, with an estimated ejection fraction (EF) of 20%. The patient had multiple LV wall abnormalities including akinetic inferolateral and apical anterior walls and hypokinetic basal and septal walls. A small pericardial effusion was also noted (Figure 2). The following differential diagnoses were entertained: acute coronary syndrome, myopericarditis, and stress-induced cardiomyopathy.

**Figure 3.**
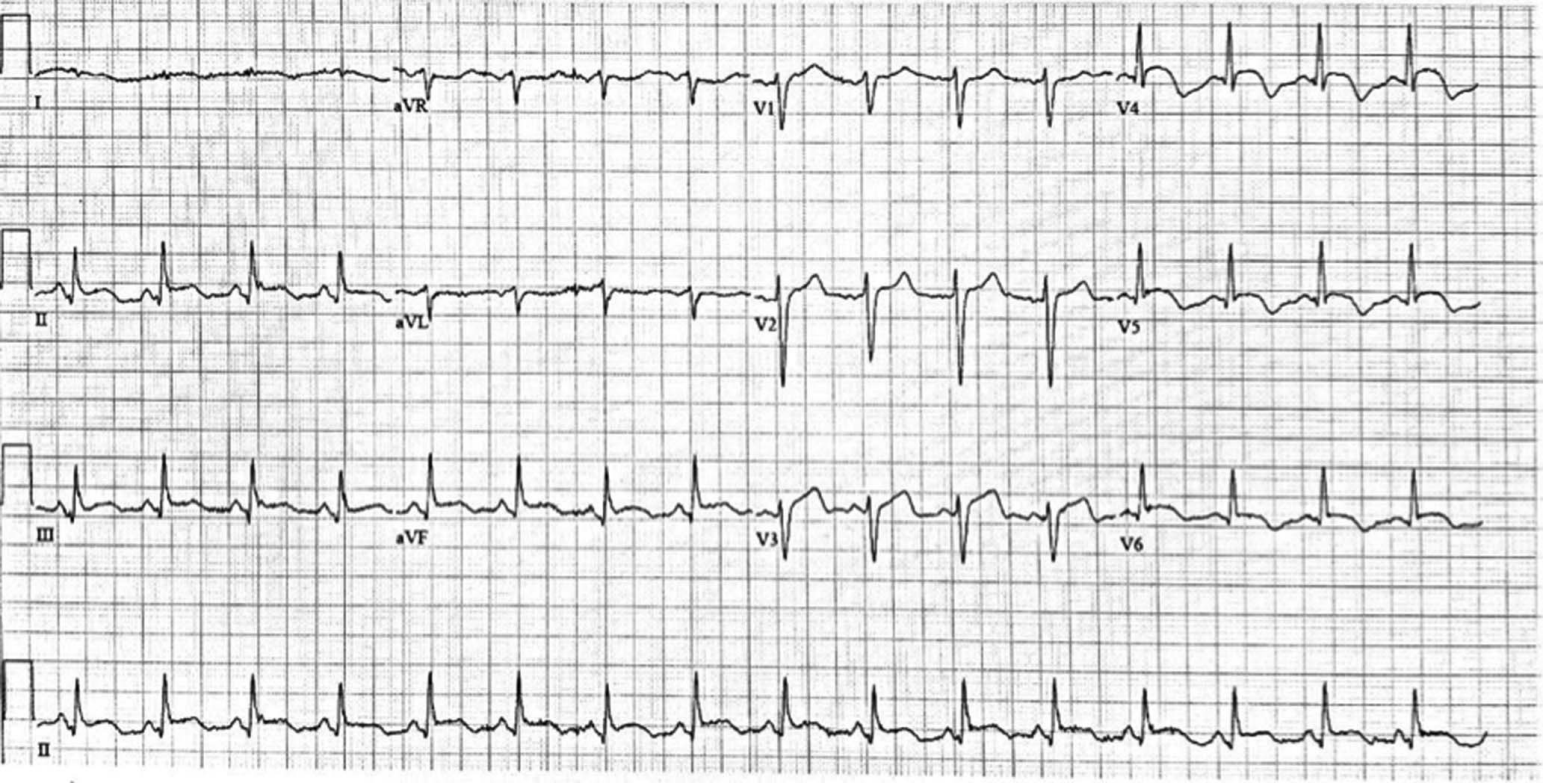
Follow-up electrocardiogram shows ST elevations.

On hospital day 4, the patient became markedly agitated, disoriented, and confused. Physical examination revealed normal heart sounds without murmurs, gallops, or rubs, but the patient was noted to have jugular vein distention, decreased breath sounds, lower extremity swelling, and cool extremities. Laboratory tests revealed leukocytosis with a white blood cell count of 30 K/uL (reference range, 3.9-12.7 K/uL), elevated lactic acid at 4.2 mmol/L (reference range, 0.5-2.2 mmol/L), increased CRP of 71.9 mg/L, ferritin of 394 ng/mL (reference range, 20-300 ng/mL), LDH of 591 U/L, and B-type natriuretic peptide of 2,336 pg/mL (reference range, 0-99 pg/mL). Meanwhile, cardiac troponin had trended down from 1.0 to 0.2 ng/mL. Computed tomography scan of the head without contrast was negative for ischemia or intracranial hemorrhage. Repeat ECG noted new diffuse ST elevations, most prominent in the anterolateral and inferior leads and with persistent ST elevation in the inferior leads (Figure 3). Central venous catheter was placed and showed low cardiac output with a significantly reduced mixed venous O_2_ saturation of 28%. Coronary angiography was not performed because of the patient's infectious state and clinical instability.

Because of the patient's hypotension, low output state, and congestion, she was transferred to the intensive care unit (ICU) and treated with dobutamine infusion at an initial rate of 2.5 μg/kg/min (up-titrated to 5 μg/kg/min), inhaled nitric oxide at 10 ppm, norepinephrine infusion (0.02-3.0 μg/kg/min), and furosemide 20 mg/h infusion. Heparin infusion and ticagrelor were discontinued because of low suspicion for acute coronary syndrome at that point. The patient's antibiotic regimen was also adjusted, and IV vancomycin 1,250 mg every 24 hours and piperacillin/tazobactam 4.5 g every 8 hours were added (Table 1). Her ICU course was further complicated by tachyarrhythmias, including atrial fibrillation with rapid ventricular response and ventricular tachycardia, which resolved after the addition of amiodarone infusion (150 mg to start, followed by 1 mg/min continuous infusion).

**Table 1.** Medication Details

Medication	Dose and Frequency	Method of Administration	Start Day	End Day
Ceftriaxone	1 g every 24 h	Intravenous	Day 1	Day 4
Azithromycin	500 mg once, then 250 mg daily	Oral	Day 1	Day 4
Methylprednisolone	125 mg once	Intravenous	Day 1	Day 1
Prednisone	40 mg every 24 h	Oral	Day 2	Day 4
Aspirin	325 mg once	Oral	Day 2	Day 2
Ticagrelor	180 mg once, then 90 mg every 12 h	Oral	Day 2	Day 4
Heparin	60 U/kg to start, followed by 12 U/kg/h continuous infusion	Intravenous	Day 2	Day 4
Dobutamine	2.5 μg/kg/min to start, followed by 5.0 μg/kg/min continuous infusion	Intravenous	Day 4	Day 12
Inhaled nitric oxide	10 ppm	Inhalation	Day 4	Day 6
Norepinephrine	0.02-3.0 μg/kg/min	Intravenous	Day 4	Day 8
Furosemide	20 mg/h continuous infusion	Intravenous	Day 4	Day 12
Vancomycin	1,250 mg every 24 h	Intravenous	Day 4	Day 6
Piperacillin/tazobactam	4.5 g every 8 h	Intravenous	Day 4	Day 6
Amiodarone	150 mg to start, followed by 1 mg/min continuous infusion	Intravenous	Day 5	Day 5
Colchicine	0.6 mg daily	Oral	Day 8	Day 14

On hospital day 6, the patient's hemodynamic status had improved. Her vasopressor requirements decreased, and she was successfully weaned off nitric oxide. Bedside TTE revealed apical wall hypokinesis but an improved LV systolic function with an EF of 35% to 40%. All antibiotics were discontinued because of the absence of evidence for bacterial infection.

On hospital day 8, the patient had been weaned off nor-epinephrine and was started on colchicine 0.6 mg daily for myopericarditis. On examination, she was alert and able to answer yes/no questions but had frequent episodes of delirium.

**Figure 4.**
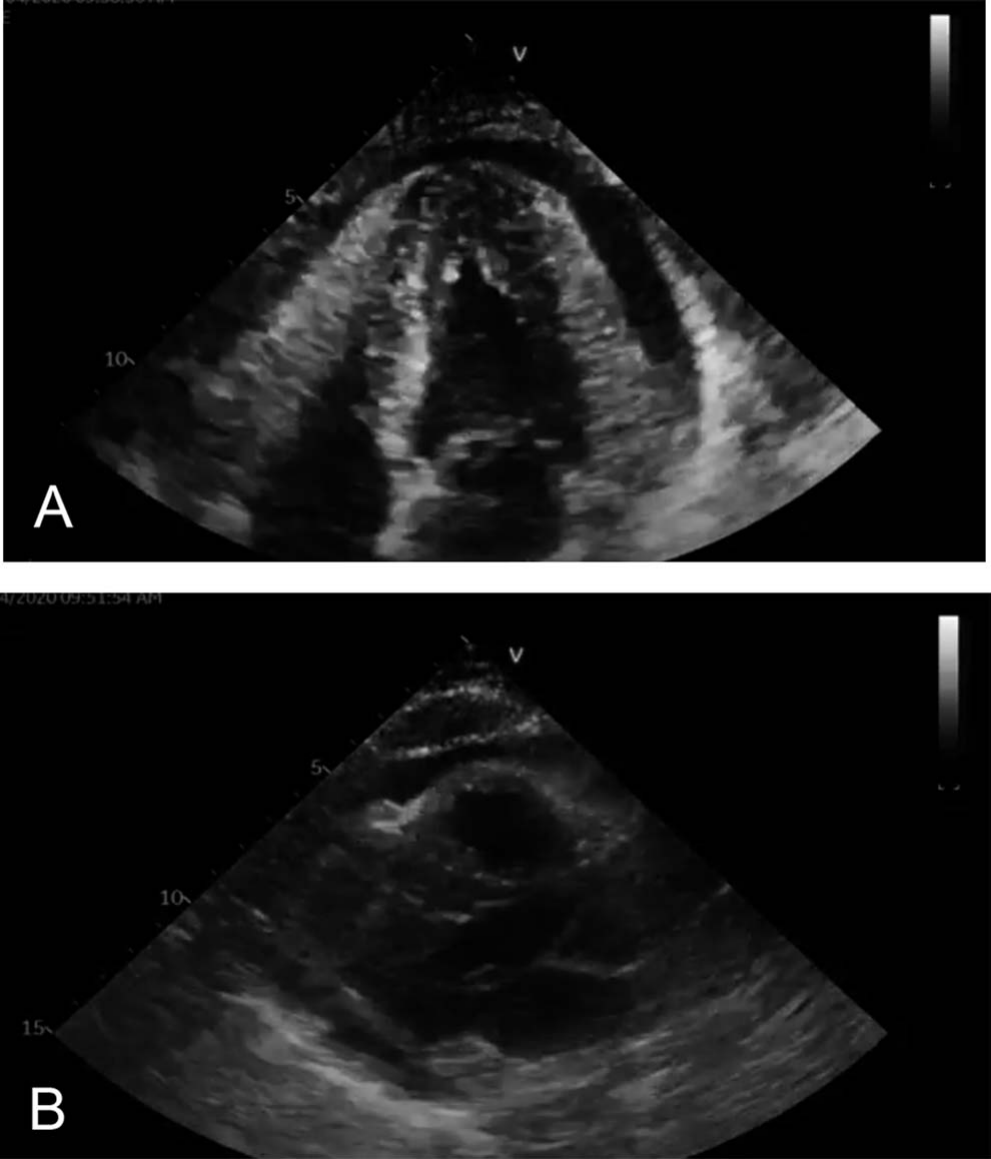
Follow-up echocardiogram in (A) 4-chamber view and (B) parasternal long axis view shows improved ejection fraction of 50%, moderate pericardial effusion, and recovery of segmental wall motion abnormalities.

Repeat ECG on day 9 showed almost full recovery of her LV function, with EF improved to 50%. Moderate circumferential pericardial effusion without tamponade physiology was noted (Figure 4).

On hospital day 12, the patient's cardiogenic shock had resolved, and she had been weaned off dobutamine. On examination, she was afebrile with a stable blood pressure of 145/65 mmHg, heart rate of 65/min, and O_2_ saturation of 98% on 2 L of O_2_ via nasal cannula. Because of the patient's significantly improved clinical status, she was transferred out of the ICU.

On hospital day 14, her clinical status deteriorated again, with worsening mentation and significant abdominal pain. Abdominal x-ray revealed signs of bowel obstruction. The patient's family was contacted, and the decision was made not to pursue further intervention. The patient was transitioned to comfort care and died shortly afterward. Table 2 presents a timeline summary of the case.

**Table 2.** Summary of Patient's Hospital Course

Day 1	Day 2	Days 3-5	Days 6-7	Day 8	Day 9	Days 10-12	Day 14
Patient admitted for hypoxic respiratory failure due to COVID-19.	Patient develops chest pain with troponin peak of 1.0 ng/mL. T wave inversions on ECG.	TTE shows EF of 20%. Diffuse ST elevations on ECG. Patient has altered mental status and signs of cardiogenic shock. Patient on vasopressor and inotropic support. Admitted to ICU.	Patient improves. Weaned from hemodynamic support. TTE shows EF of 35% to 40%.	Weaned from all vasopressor support. Started on colchicine.	TTE shows EF of 50%.	Cardiogenic shock resolved. Patient stepped down to floor.	Clinical status deteriorated; signs of bowel obstruction. Patient transitioned to comfort care.

ECG, electrocardiogram; EF, ejection fraction; ICU, intensive care unit; TTE, transthoracic echocardiography.

## DISCUSSION

During her hospitalization, a patient with acute on chronic respiratory failure in the setting of COVID-19 developed suspected acute myopericarditis with severe LV dysfunction. Although the incidence of myocarditis in patients with COVID-19 remains unknown, increasing evidence links COVID-19 to cardiovascular complications such as arrhythmias, heart failure, cardiogenic shock, fulminant myocarditis, and cardiac death.^2^

Myopericarditis presents with a variety of symptoms ranging from dyspnea and chest pain to cardiogenic shock, as observed in our case. Cardiac biomarkers troponin I and troponin T may be elevated in cases of myocarditis.^3^ Incidentally, elevated serum cardiac biomarkers have been recognized in patients with COVID-19, with significantly higher values in patients admitted to the ICU.^4^ The significance of elevated troponins, however, requires careful consideration, as elevated troponins are a nonspecific marker of myocardial injury. ECG findings with myocarditis are also nonsensitive and nonspecific, with findings such as T wave inversions, nonspecific ST segment changes, and ST elevations mimicking myocardial ischemia as observed in our case (Figure 2). In the presence of elevated cardiac biomarkers and ST elevations on ECG, acute coronary syndrome should always be ruled out. In our patient, however, the diffuse ST elevations with absence of reciprocal changes on ECG, in addition to the presence of elevated CRP, leukocytosis, and pericardial effusion on TTE, was more supportive of myopericarditis. Because of the health care risk associated with the COVID-19 pandemic and the clinical instability of this patient, endomyocardial biopsy, cardiac magnetic resonance imaging, and diagnostic coronary angiogram were not performed.

Another potential etiology was stress-induced cardiomyopathy, also known as Takotsubo cardiomyopathy.^5^ This clinical syndrome is characterized by LV dysfunction attributable to significant emotional or physical stress. Takotsubo cardiomyopathy is also characterized by apical ballooning or apical hypokinesia, akinesia, or dyskinesia, with basal hyperkinesis in the absence of major coronary disease on angiography.^5^ In our case, the TTE showed diffuse wall motion abnormalities, including basal segments, a finding that is atypical in Takotsubo cardiomyopathy.^5^ Nevertheless, in the absence of angiographic evidence, Takotsubo cardiomyopathy could not entirely be ruled out.

Several mechanisms have been suggested in the pathogenesis of COVID-19–related myocardial injury. One is via direct myocardial lysis, where the virus gains entry into the cardiac myocyte by binding to the angiotensin-converting enzyme-2 (ACE2) receptor, leading to changes in the ACE2 pathway with consequent myocardial injury.^2,5^ Alternatively, elevated proinflammatory cytokines, commonly observed in patients with COVID-19, may trigger an exaggerated response from the immune system, leading to myocardial injury.^2,6^ Myocardial injury can also result from the body's inability to match the myocardial O_2_ demands in the setting of increased metabolism caused by systemic infection from COVID-19 and hypoxemia from pneumonitis/pneumonia/acute respiratory distress syndrome.^2^ Finally, given the high-stress state associated with an acute illness such as COVID-19, catecholamine surge and systemic inflammation may lead to plaque destabilization, rupture, and, eventually, acute coronary syndrome.^5^

Given the multiple mechanisms of myocardial injury in patients with COVID-19, consideration of targeted therapeutic options is essential. Data published in October 2020 suggest some benefit with the use of corticosteroids in COVID-19 patients with myocarditis.^7^ Our patient was treated with steroids in the context of acute respiratory failure and COPD exacerbation, in addition to colchicine for myopericarditis. Colchicine is a well-known anti-inflammatory drug used in the treatment of pericarditis.^8,9^ A 2019 paper by Tardif et al showed that colchicine is beneficial in reducing adverse cardiovascular events.^10^ The use of glucocorticoids and colchicine in COVID-19 patients with myopericarditis may have some benefit.

## CONCLUSION

This case highlights the diagnostic and therapeutic challenges that physicians may encounter when managing acute cardiac injury in the setting of COVID-19. We believe it is essential to recognize that the multiple mechanisms of COVID-19–related myocardial injury may influence the approach to diagnosis and treatment. Also, further evidence is needed to determine the utility of immunomodulatory agents and glucocorticoid therapy for patients with suspected COVID-19 myocarditis.

## Notes

*This article meets the Accreditation Council for Graduate Medical Education and the American Board of Medical Specialties Maintenance of Certification competencies for Patient Care and Medical Knowledge.*
